# Supplementary material for: Heart Rate Variability Measurements Across the Menstrual Cycle and Oral Contraceptive Phases in Two Olympian Female Swimmers: A Case Report
Source: Sports (Basel). 2025 Jun 12;13(6):185. doi: 10.3390/sports13060185 (PMC12197002; doi:10.3390/sports13060185)
Supplement: Supplementary file 1 [file sports-13-00185-s001.zip › sports-3655677-supplementary.pdf]

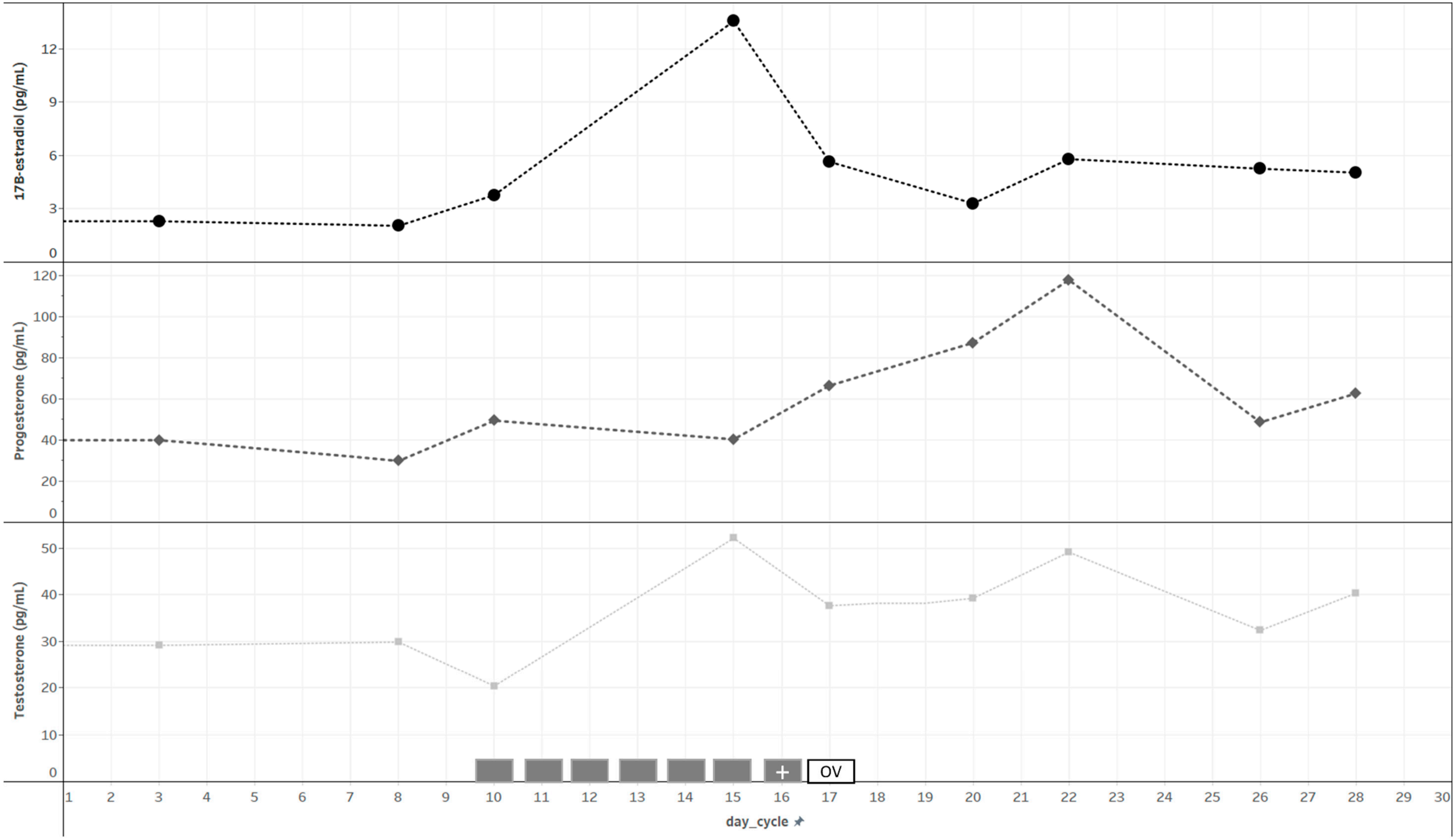

Figure S1: Data collected from salivary samples for Athlete 1's third cycle. OV: ovulation day. Grey box: urinary ovulation tests. +: luteinizing hormone surge.

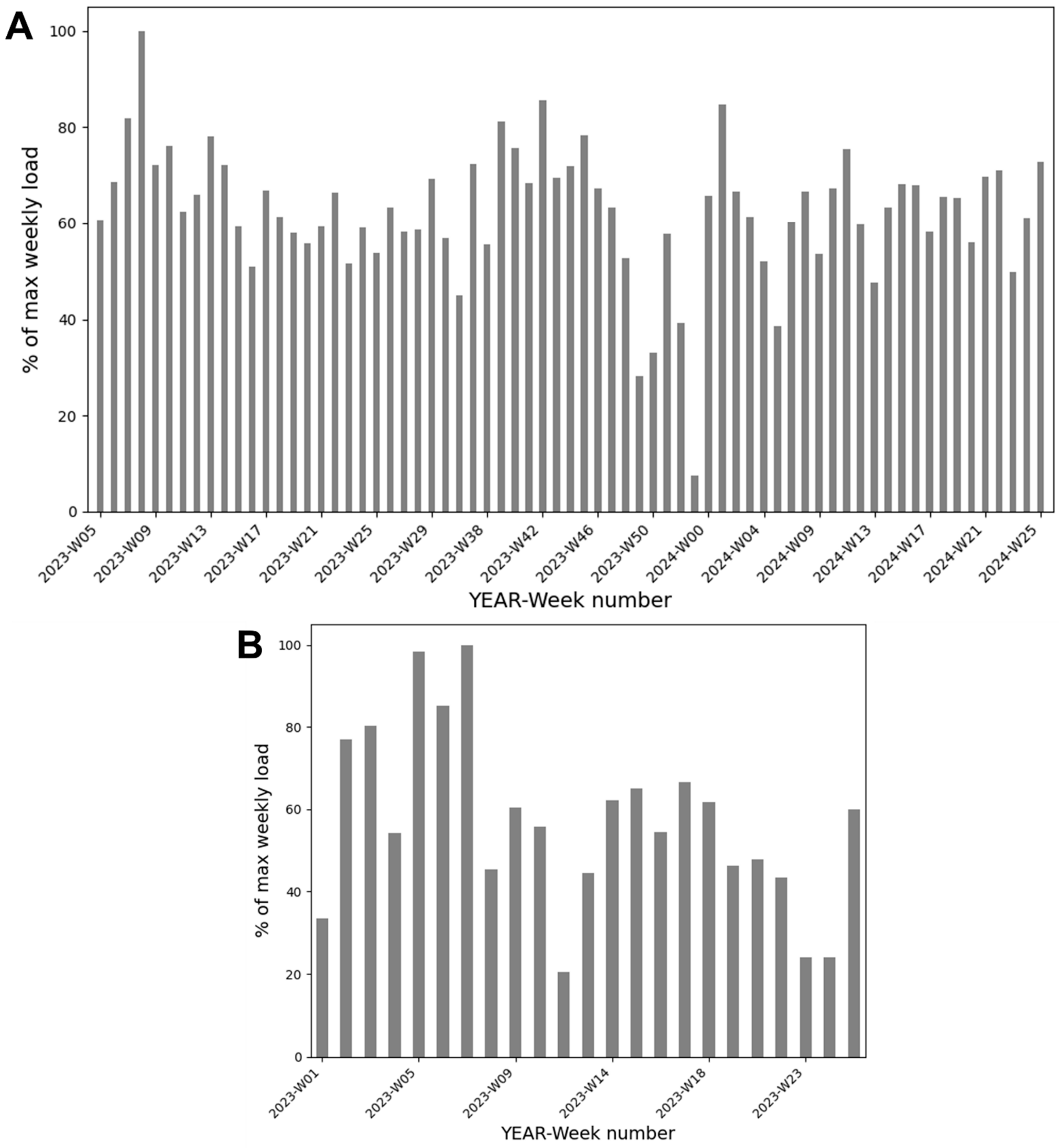

Figure S2: Weekly training load as percentage of maximum week for Athlete 1 (A) and Athlete 2 (B).
